# Gating, enhanced gating, and beyond: information utilization strategies for motion management, applied to preclinical PET

**DOI:** 10.1186/2191-219X-3-29

**Published:** 2013-04-24

**Authors:** Adam Leon Kesner, Galith Abourbeh, Eyal Mishani, Roland Chisin, Sagi Tshori, Nanette Freedman

**Affiliations:** 1 Department of Medical Biophysics and Nuclear Medicine, Kiryat Hadassah, P.O. Box 12000, Jerusalem 91120, Israel; 2 Cyclotron/Radiochemistry Unit, Hadassah University Hospital, Kiryat Hadassah, P.O. Box 12000, Jerusalem 91120, Israel

**Keywords:** Motion control, Software-based gating, Hardware-based gating, Gating+, Inter-gate values, Small-animal PET

## Abstract

**Background:**

Respiratory gating and gate optimization strategies present solutions for overcoming image degradation caused by respiratory motion in PET and traditionally utilize hardware systems and/or employ complex processing algorithms. In this work, we aimed to advance recently emerging data-driven gating methods and introduce a new strategy for optimizing the four-dimensional data based on information contained in that data. These algorithms are combined to form an automated motion correction workflow.

**Methods:**

Software-based gating methods were applied to a nonspecific population of 84 small-animal rat PET scans to create respiratory gated images. The gated PET images were then optimized using an algorithm we introduce as ‘gating+’ to reduce noise and optimize signal; the technique was also tested using simulations. Gating+ is based on a principle of only using gated information if and where it adds a net benefit, as evaluated in temporal frequency space. Motion-corrected images were assessed quantitatively and qualitatively.

**Results:**

Of the small-animal PET scans, 71% exhibited quantifiable motion after software gating. The mean liver displacement was 3.25 mm for gated and 3.04 mm for gating+ images. The (relative) mean percent standard deviations measured in background ROIs were 1.53, 1.05, and 1.00 for the gated, gating+, and ungated values, respectively. Simulations confirmed that gating+ image voxels had a higher probability of being accurate relative to the corresponding ungated values under varying noise and motion scenarios. Additionally, we found motion mapping and phase decoupling models that readily extend from gating+ processing.

**Conclusions:**

Raw PET data contain information about motion that is not currently utilized. In our work, we showed that through automated processing of standard (ungated) PET acquisitions, (motion-) information-rich images can be constructed with minimal risk of noise introduction. Such methods have the potential for implementation with current PET technology in a robust and reproducible way.

## Background

In the evolution of nuclear medicine imaging technologies, there have been steady advancements towards better sensitivity and resolution. Resolution has in fact improved so much that for some parts of the body, further improvements will be of no help as respiratory and cardiac motions limit the benefits. Respiratory and cardiac motions cause blurring in imaging, particularly around the lungs and diaphragm [1,2]. Image degradation may include poorer lesion detectability and inaccuracy in location, volume definition, and quantitation. Gating may be used to overcome these problems and realize the benefits of high-resolution imaging [3-5].

Respiratory gating has been studied for over a decade in PET imaging, both in small-animal PET [6] and human PET [7,8]. Many commercial systems today include hardware devices, e.g., a pressure belt [9-11], motion camera [8,12], or other systems [13,14], to monitor respiratory signal, along with gating software to process it. However, in the past few years, several data-driven algorithms have been presented for extracting respiratory signal directly from the raw scan data without using hardware. These algorithms perform comparably to hardware [12,15], can be fully automated [16-19], and can be used with no changes to current clinical scanning procedures.

Software-based algorithms can offer advantages over hardware-based gating, both in preclinical and clinical environments. Since they are based solely on analysis of image data and not hardware equipment, they can be used with existing scanners. In contrast to hardware systems, they avoid a possible source of subject discomfort, costly equipment, and potential for equipment failure. They require no additional scan setup time or staff training and avert higher radiation doses to patients and technologists from the added operations/slower throughput [20]. Such methods are operator independent and reproducible, and the gating signals are intrinsically aligned with the image data.

Beyond gated image acquisition, questions arise as to how to best use the data. While conventional PET produces summed images of all phases of the respiratory cycle, gating generates a series of images at different phases of the cycle. These gated images have improved spatial resolution since each image includes only a short phase of the respiratory cycle, but each image also includes fewer counts than the summed ungated image and, thus, is noisier. Because of this inherent trade-off between noise and resolution, it can be difficult for the human eye, or even computer-aided systems, to distinguish between added value (e.g., organ edges, motion) and misleading information introduced by noise effects [10].

Techniques have been developed to address the sacrifice in statistics inherent in gating. While strategies for optimized data binning have been presented [11,21], the primary efforts for full data utilization use nonlinear deformation maps to map information from different gates to a target gate, essentially recombining the gates back into a single motion-free frame with high resolution and high count statistics [22-24]. Limitations of these techniques are that they can be complex, parameter and distribution dependent, prone to error, difficult to fully characterize, and some require heavy processing [25,26].

A very different approach used previously to reduce noise in gated nuclear medicine data is through filtering. Temporal frequency filters can be applied to dynamic or gated data to produce less noisy images [27]. Filtering offers a computationally easy method for reducing noise but has not been adopted for larger-scale use. One problem of the technique is that filtering may improve the accuracy in some pixels but not in others. Voxel-specific filtering, based on the noise and signal characteristics of each individual voxel, has the potential to avoid this problem [28].

In recent years, methods based on strategies of randomly sorting or bootstrapping data have been developed to estimate effective gate-specific, voxel-specific noise in gated data [29]. This approach provides a measure of effective noise, essentially independent of the processing routines used to create the image. The method we propose in this paper utilizes this concept for noise estimation and combines it with previously presented ideas for noise filtering as a foundation for developing an advanced filtering technique for optimizing information in the four-dimensional (4D) (gated) signal.

In the work presented here, we aim to extend and improve a strategy for creating software-gated images, previously applied to human PET, to small-animal PET using a population of scans acquired with a variety of radiotracers. In addition, we present a new voxel-based filtering approach, which we denote as ‘gating+’, to address the problem of low statistics in subsampled gated images and to enable clear visualization of image features in the presence of respiratory motion. Validation is performed using simulations as well as the small-animal PET scans.

## Methods

Software-based respiratory signal was extracted from the raw listmode files of 84 rat PET scans. The data consisted of all scans with duration of at least 10 min acquired during research studies at our institution over a period of 12 months. All studies were approved by the Animal Research Ethics Committee of the Hebrew University of Jerusalem. Tracers utilized included ^18^F-fluorodeoxyglucose (^18^F-FDG, *n* = 27), ^11^C-dimethyl-diphenyl-ammonium (^11^C-DMDPA, *n* = 10) [30], ^13^N-NH_3_ (*n* = 5), ^11^C-choline (*n* = 2), ^18^F-NaF (*n* = 1), ^18^F-fluoroethyl-diphenyl-methyl-ammonium (^18^F-FEDPMA, *n* = 12) and ^18^F-fluorobuthyl-diphenyl-methyl-ammonium (^18^F-FBDPMA, *n* = 4) (^18^F-FEDPMA and ^18^F-FBDPMA are investigational new compounds for PET myocardial perfusion imaging), and ^18^F-ML10 (*n* = 23, agent for imaging apoptosis [31]). Twenty four of the scans (11 ^18^F-FDG, 5 ^11^C-DMDPA, 4 ^18^F-FEDPMA, and 4 ^18^F-FBDPMA) were acquired with hardware-based respiratory gating, and for this subset of scans, hardware-based signals were compared with the corresponding software-based signal.

### Scan acquisition

All scans were acquired using a Siemens Inveon small-animal PET scanner (Siemens Healthcare®, Knoxville, TN, USA). Scans were reconstructed, using three-dimensional (3D) sinograms and OSEM2D reconstruction (4 iterations and 16 subsets), into 128 × 128 × 159 images with a voxel size of 0.7764 × 0.7764 × 0.796 mm^3^. All images were smoothed with a 2-mm^3 ^full width at half maximum (FWHM) Gaussian smoothing filter. Random correction was performed by subtraction of delayed coincidences. No attenuation or scatter corrections were used. For the 24 scans acquired with hardware-based gating, the gating signal was acquired using a Biovet® gating system (Biovet®, M2Mimaging, Cleveland, OH, USA). This system acquires respiratory signal through the use of a pressure-sensitive pad placed beneath the rat.

The duration of scanning differed among the research studies. For the sake of uniformity, only 10 min of each scan was analyzed. The 84 scans were acquired using rats of varying strains and sizes (100 to 500 g) in prone position, with injected doses ranging between 300 μCi and 2 mCi, and total detected prompts ranging between 4.8 × 10^6 ^and 7.9 × 10^8 ^counts (over 600 s). The part of the animal included in the 126.6-mm axial field of view (FOV) varied since the 84 scans were from diverse studies, with organs of interest ranging from the brain, the heart, the lungs, or leg muscles; thus, while most scans included all or almost all of the thorax and abdomen, some did not.

### Software-based gating procedures

Listmode files, the initial raw output from a PET scanner, consist of a list of detected events interspersed by time stamps. In the case of hardware gating, the listmode file includes hardware-based gating triggers inserted at relevant time points; these triggers are used during construction of the gated images.

The main steps involved in data-driven gating can be summarized as follows:

Step 1. Respiratory signal is extracted from the listmode data.

Step 2. Respiratory gating triggers, derived from step 1 signal, are inserted to form a new *gated* listmode file (analogous to the one created with hardware-based triggers).

Step 3. Gated images are reconstructed from the new listmode file.

The software-based gating method has been described previously for application in human PET [18,19]. As a subject breathes, the activity concentration in fixed regions of space fluctuates with frequencies corresponding to respiration. The signal in each of these regions is small and noisy, but by combining the fluctuating signal in many regions, we can extract a useful ‘global’ respiratory signal to be used for gating. The algorithm was implemented as described previously but with relevant parameters adjusted for differences across the technologies/species. The average breathing frequencies for humans and rats are approximately 0.2 and 1 Hz, respectively. Accordingly, the frequency pass window we used here for voxel prioritization and combination was 0.66 to 3.33 Hz. The time bin parameter - duration of the short time sinograms used for sampling time activity - was 100 ms, approximately one-tenth of an average respiratory cycle [18,32]. Finer sampling times were deemed unnecessary in this step, particularly in light of the fact that the vendor supplied gating software utilized moving averages (over 8 cycles) for respiratory period determination.

In addition to the published methods, one enhancement was introduced: Previously, when the signal from each sinogram region was combined with the global signal, a simple test was applied to see if the signal was in phase or out of phase with the global signal (step 5B in previous article [18]). This same test was presented here, but the entire signal duration was split into multiple (six) equal time segments (i.e., time-activity curves with length of 10 min / 6). The test was applied to all six segments separately, and only when all phase tests agreed was the signal of a small sinogram region added to the global respiratory signal. This modification helped exclude data from voxels that were too noisy or did not contain useful information, thus improving the signal-to-noise ratio in the output. The value of six segments was chosen empirically to be great enough to consistently filter out random signals while not so great that it would filter out useful signal. As a clarification, uniform periodicity is not a requisite for this technique to adequately capture respiratory signal.

In our specific implementation, 3D data were binned into two-dimensional (2D) sinograms using single-slice rebinning (SSRB) [33] (a strategy used for data-driven gating presented by Schleyer and colleagues [12]). The SSRB sinograms had dimensions (*ρ*, *θ*, *z*) of 128 × 160 × 159. The voxels had dimensions *ρ* = 0.815 mm and *θ* = *π* rad/160, and the scanner had an axial crystal pitch of 1.592 mm.

In step 2, the one-dimensional (1D) global respiratory trace acquired in step 1 was analyzed to extract respiratory triggers. The trigger points, i.e., times where a new respiratory cycle has begun, were defined as points in time when the 1D global respiratory trace was at a local maximum (±1/2 the most represented respiratory period (s)). These respiratory trigger points were then inserted back into the raw listmode files (in the format suitable for our reconstruction software) to create modified listmode files.

In step 3, 4D gated image data sets were generated from the listmode files using the Siemens reconstruction software exactly as they would have been reconstructed in the case of hardware gating. The 4D gated data sets contain information in three spatial dimensions and one temporal (i.e., gated) dimension. In this paper, we will refer to the dimension of information across gates as ‘temporal’.

### Gating+ algorithm

Gating+ uses a frequency filter to generate enhanced gated images that include respiratory motion but preserve the favorable noise characteristics of the ungated image. The filter passes the ungated image (zero frequency) plus those higher frequencies that add more (constructive) motion information than (destructive) noise. Noise and motion are nonuniformly distributed throughout an image; correspondingly, we used the signal and noise characteristics of each individual voxel to generate a scan-specific and spatially variant band-pass filter.

Voxel-specific noise characteristics are evaluated using a method of random gating. A 4D randomly gated image set is generated by subdividing the original listmode data based on random triggers instead of triggers derived from respiratory motion. The behavior of the resultant images provide an indication of how much noise and fluctuations are to be expected simply from the act of gating, without any motion information confounding the issue. The voxel-specific noise reflects statistical noise in subsampled signal, random fluctuations, and any system noise/bias. Characterized for our processing, a voxel's *effective noise* magnitude is conservatively defined as the maximum signal amplitude of all nonzero frequencies in the randomly gated data set.

**Figure 1:**
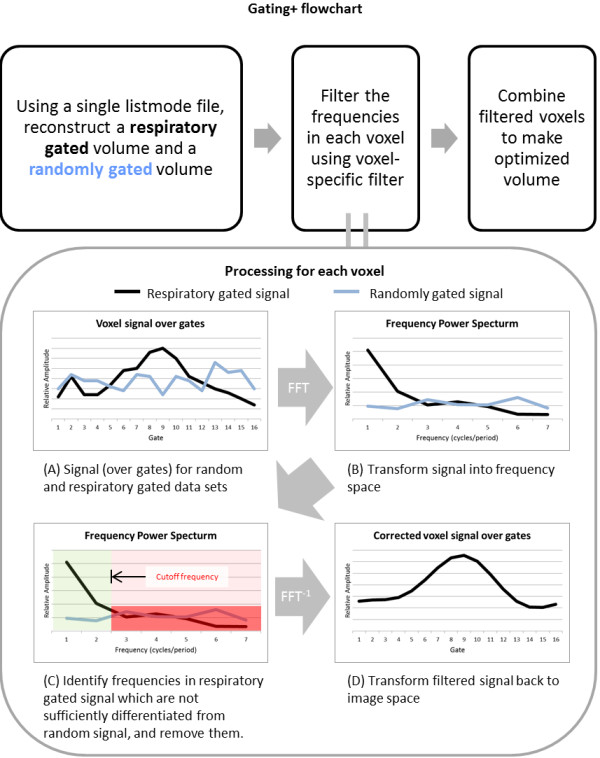
**Flowchart illustrating gating+ workflow.** The processing applied to each voxel is shown in the lower box. ‘Respiratory gated signal’ and ‘randomly gated signal’ refer to the signals for the same voxel in the respiratory motion gated and randomly gated datasets, respectively.

To implement voxel-specific filtering, the fast Fourier transform is first applied to each voxel in both the respiratory gated and the randomly gated data sets in the temporal dimension. This yields two sets of vectors in real and imaginary frequency space, describing both motion signal and effective noise in the case of the respiratory gated data, and effective noise alone in the case of the randomly gated data. The gating+ algorithm compares the vector magnitudes at every frequency to determine the appropriate frequencies to pass - effectively those frequencies that make a discernible contribution. Because higher frequencies require greater statistics to support them, the useful-frequency windows span lower frequencies to an upper cutoff frequency. Specifically, a band-pass filter is defined with a lower bound of 0 (direct current (DC) signal), and an upper bound determined as the highest contiguous frequency where the magnitude of the fluctuating signal is sufficiently greater than the magnitude of the noise (>1.2 × effective noise magnitude for the corresponding voxel in the randomly gated dataset). The gated information in that frequency window is allowed to pass through the filter and thus modify the image from its ungated embodiment (zero frequency image). Signal in frequencies outside this window are filtered, i.e., truncated to 0. The threshold of 1.2 was derived from Monte Carlo simulations which address the fact that noise presents with an unknown/random phase (see ‘Discussion’ section for further details). This value represents the probability threshold where using the gated signal fluctuations becomes advantageous (*P*(1.2) = 0.5). After all voxels are processed, gating+ images are obtained by performing the inverse fast Fourier transform on the filtered data to yield a set of enhanced gated images. A flowchart illustrating the gating+ process is shown in Figure 1.

### Motion maps and inter-gate phase shifting

**Figure 2:**
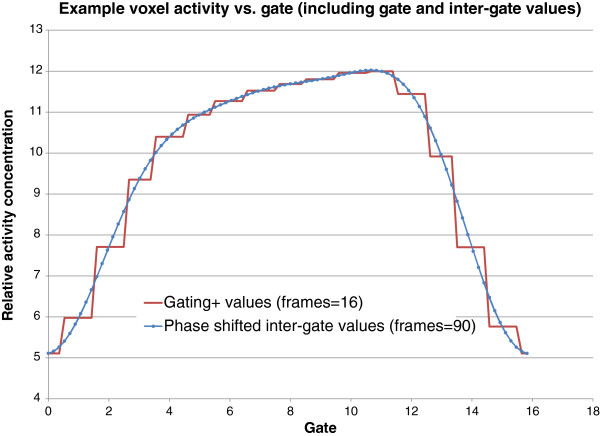
**Example voxel activity curve.** Taken from rat FDG μPET image (16 gating+ gates, one respiratory cycle). Gating+ (gated) values presented with corresponding phase-shifted inter-gate values. Image illustrates a high correlation of gate values with inter-gate values.

In addition to optimizing the signal, the filtering of noise in the temporal domain also provides two interesting opportunities. Firstly, the frequency pass map, constructed during gating+ filtering, can provide an overview of the detected motion - a ‘motion map’. Secondly, because signal is optimized in frequency space where it is not bound to gates it was created with, there is a particular opportunity to manipulate its real and imaginary components to achieve a phase shift when it is transformed back into image space, essentially allowing us to extract inter-gate voxel values (demonstrated in Figure 2). By uniformly shifting all voxels, we can reconstruct phase-shifted images that may correspond to any or all phases of the motion cycle. In the work here, we used this process to create ‘continuous motion image’ (CMI) sequences with finely timed frames that span the motion cycle. The CMIs offer an alternative visualization of motion and may present a new platform for assessing 4D data.

### Validation

To assess the accuracy of gating+ images, simulations were generated, consisting of 2D + time images containing a hot lesion set in a colder background moving adjacent to a stationary two-compartment structure, with varying amounts of random noise added to simulate environments of varying signal statistics. For the PET data, each listmode file was used to reconstruct an ungated image, gated images, gating+ images, motion map, and CMIs. All images were reconstructed with 16 gates and CMIs with 90 frames. The large number of gates was chosen because, in contrast to gating, gating+ images appeared to benefit from having more gates, i.e., more available frequencies to utilize when statistics support it.

Gated PET images were rendered in the form of maximum-intensity projection image sequences for qualitative review by four independent reviewers who evaluated the scans for presence of ‘obvious characteristics of respiratory motion’. For quantitative comparison of hardware-gated, software-gated, and gating+ images, we used the global center of mass (COM) displacement as a general measure of motion.

Many aspects of respiratory motion will shift the global activity COM during the respiratory cycle, although it is acknowledged that some do not. The COM displacement between gates appeared to provide a useful, albeit imperfect, measure of respiratory motion that can be applied to any image. The COM was calculated for each gate (Equation 1):

(1) $$x_{\mathrm{cm}}\;=\;\frac{\sum_{i}m_{i}x_{i}}{M}\;y_{\mathrm{cm}}\;=\;\frac{\sum_{i}m_{i}y_{i}}{M}\;z_{\mathrm{cm}}\;=\;\frac{\sum_{i}m_{i}z_{i}}{M}$$

where *m*_i _is the summed voxel intensity for the image plane, Σ_i_ is the sum of all image planes in the respective dimensions, *x*_i_, *y*_i_, and *z*_i_ are the coordinates of the image planes, *M* is the total activity in the images, and *x*_cm_, *y*_cm_, and *z*_cm _are the coordinates of the COM. The maximum COM displacement was calculated as the greatest distance (magnitude of the displacement vector) between any two COMs from different gates. Maximum COM displacement was calculated for all hardware-gated, software-gated, and gating+ scans. In order to justify its validity with respect to our data, we also calculated for global COM measurements for the randomly gated scan set for comparison.

In addition to tracking motion using the COM, we also used volume of interest (VOI) and line profile analysis for those scans that had certain definable characteristics. For scans with uptake in the kidneys, 0.016-cc (33 voxels) spherical VOIs were placed at each kidney centered on the maximum-intensity pixel of an additionally smoothed image (Gaussian smoothing kernel, FWHM = 0.5 cm^3^). For scans with uptake in the liver, line profiles were manually placed to characterize the lung/liver boundary. Background noise levels were assessed in all 84 datasets by manually placing a 0.2-cc (approximately 400 voxels) spherical volume of interest in low-uptake areas of the shoulder, or the hip region in cases where no shoulder was available.

## Results

### Software and hardware comparison

The 24 scans that had been constructed using both hardware- and software-based respiratory triggers were assessed and compared (Table 1). The median respiratory periods measured from hardware and software triggers were within 5% of each other for 22/24 scans, ±5% being the envelope that can be expected due to the difference in timing resolutions: software at 100 ms and hardware at 10 ms.

**Table 1:** **Hardware-vs.-software compariso**n

**Scan number**	**Events in scan (trues), ×10**^**8**^	**Median respiratory period (s)**			**Maximum COM displacement (mm)**			**Motion observed by all four independent observers**
		**Software**	**Hardware**	**Difference**	**Software**	**Hardware**	**Difference**	
1	1.4	1.00	0.98	0.02	1.16	1.20	−0.04	Yes
2	1.4	1.20	1.23	−0.03	0.47	0.33	0.14	Yes
3	1.6	1.10	1.06	0.04	0.96	0.91	0.05	Yes
4	1.6	1.20	1.21	−0.01	1.37	1.89	−0.52	Yes
5	1.6	1.30	1.26	0.04	0.28	0.37	−0.09	Yes
6	1.9	1.10	1.07	0.03	1.48	1.40	0.08	Yes
7	2.1	1.20	1.22	−0.02	0.19	0.21	−0.02	Yes
8	2.1	1.20	1.15	0.05	1.43	1.51	−0.08	Yes
9	2.3	1.10	1.12	−0.02	0.94	0.92	0.02	Yes
10	2.5	0.90	1.18	−0.28	0.26	0.16	0.10	No
11	2.6	1.20	1.23	−0.03	1.38	1.55	−0.17	Yes
12	2.6	1.40	1.35	0.04	0.21	0.20	0.01	Yes
13	2.7	1.20	1.24	−0.04	0.58	0.58	−0.01	Yes
14	2.9	1.20	1.15	0.05	1.08	1.03	0.04	Yes
15	2.9	1.10	1.06	0.04	0.34	0.32	0.02	Yes
16	3.0	1.20	1.22	−0.02	0.26	0.33	−0.07	Yes
17	3.1	1.20	1.30	−0.10	0.21	0.26	−0.05	No
18	3.1	1.20	1.17	0.03	0.35	0.37	−0.03	Yes
19	3.5	1.10	1.14	−0.04	0.19	0.23	−0.04	Yes
20	3.5	1.20	1.16	0.04	0.16	0.19	−0.03	Yes
21	3.9	1.10	1.06	0.04	1.36	1.27	0.09	Yes
22	4.2	1.20	1.16	0.04	1.49	1.51	−0.02	Yes
23	4.8	1.10	1.07	0.03	1.30	1.47	−0.17	Yes
24	4.9	1.10	1.10	0.00	1.07	1.12	−0.06	Yes

Summary of median respiratory periods and maximum COM displacement for 24 scans acquired using both software and hardware gating triggers.

### Software gating

**Figure 3:**
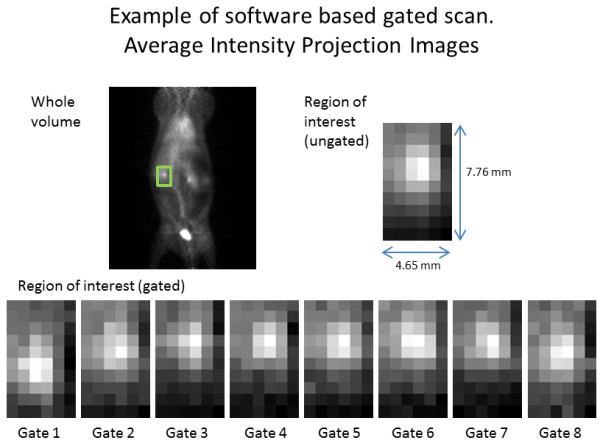
**Example software-gated image.** Projection image illustrating motion of kidney seen using software-based gating methods. Image data were acquired using a standard (ungated) acquisition.

All 84 scans were processed to yield 4D gated images. Figure 3 shows an example of a software-gated scan. The majority of software-gated images, 63/84 (75%), were confirmed to have characteristic respiratory motion as assessed by four independent reviewers.

**Figure 4:**
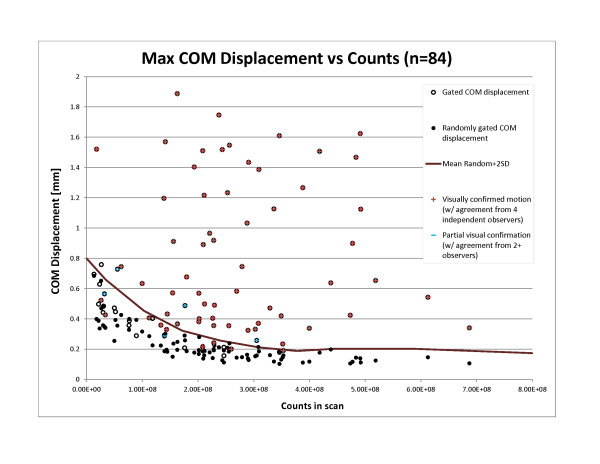
**Center of mass displacements in respiratory gated images.** Maximum center of mass displacements are displayed for the population of small-animal PET scans for both respiratory motion gated and randomly gated datasets. The moving average of the values corresponding to the randomly gated data plus two standard deviations is also displayed. This threshold was used to characterize the scans (those lying above the line) that were enhanced with motion as a result of software gating.

The maximum COM displacement values were 0.71 ± 0.46 mm (0.16 to 1.89 mm) (mean ± SD (range)) for the software-gated images. A summary of the COM data and its correlation with observer data is illustrated in Figure 4. The maximum COM data for the corresponding randomly gated scans are also shown in this figure. We observed a correlation between count statistics and random COM displacements. An envelope - defined as the moving average of the random measurements plus two standard deviations (also shown in Figure 4) - was subsequently used to characterize a count-specific threshold that delineates significant/nonsignificant displacements resulting from gating. Those software gating COM measurements lying above this cutoff (*n* = 60/84, 71%) were considered to reflect respiratory motion identified by gating. The software gating COM measurements lying below this threshold were considered to possibly be an expression of noise only. This cutoff is approximate and based on a statistical probability that approximately 97.8% of random measurements will fall below this cutoff, and is used to provide a quantitative separation between verified true respiratory motion and noise. While scans with low COM results may indicate incorrectly gated data, it is also possible that these are correctly gated scans that would not benefit from gating, an idea supported by our hardware-software comparison.

Scans with lower total counts tended to have lower COM displacement values, but there was no global correlation (Pearson correlation coefficient = 0.13). Scans that were qualitatively assessed by reviewers to exhibit motion had on average 2.6 × 10^8^ true counts, while non-motion scans had on average 59% less. Scans that had observable uptake in the heart and/or liver had a higher rate of reviewer-confirmed motion, 82% (49/60). Scans that did not have uptake in the heart nor liver, including scans that did not include this region in the FOV, had a lower rate of confirmed motion 58% (14/24).

### Gating+ gate recombination

**Figure 5:**
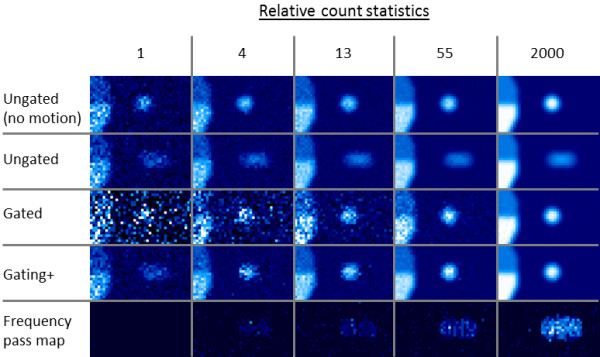
**Lesion motion simulations.** Gated simulation of a hot lesion moving in sinusoidal motion (left to right) over a colder background next to a stationary boundary. Simulated with 16 gates. The effective views show (top to bottom) count-equivalent ungated images with no motion, ungated images with motion, and a single-gate gated image, a corresponding gating+ image and a respective motion maps generated during the gating+ processing. Differing scan conditions are shown with lower count scenarios on the left, ranging to higher count scenarios towards the right. Lesion displacement = 60% lesion diameter. Lesion/background ratio = 3. Upper diaphragm/background = 1.5. Lower diaphragm/background = 3.0. Linear color scale.

Gated volumes were processed to create gating+ volumes both for the small-animal PET scans and simulations. Gating+ images consistently exhibited resolution improvement similar to that of the gated scans while maintaining lower noise levels in uniform or low-count areas. Simulations with a moving lesion are shown in Figure 5, and corresponding measurements from that data are displayed in Table 2, confirming favorable resolution and noise measurements in the gating+−processed images. Also seen in Figure 5 are the motion maps created as part of the gating+ processing.

**Table 2:** Simulation measurements

**Relative counts in image space**	**1**	**4**	**13**	**55**	**2,000**
Maximum					
Ungated	94	82	78	75	74
Gated	219	146	122	108	100
Gating+	95	125	115	108	100
Volume (70% maximum)					
Ungated	57	144	177	194	199
Gated	6	17	49	84	99
Gating+	55	41	76	89	103
SUV (mean VOI/background)					
Ungated	91	77	74	73	72
Gated	249	153	118	104	100
Gating+	92	122	110	105	100
FWHM					
Ungated	144	174	183	187	188
Gated	38	68	90	98	100
Gating+	143	88	96	94	100

Summary of lesion measurements from simulations of hot lesion against colder background with a range of random noise levels added (see Figure 5). Numbers represent average measurements from a single gate of 500 simulations for each noise scenario. All measurements are presented as a percentage of the corresponding true noise-free values. The standardized uptake value (SUV) was calculated from for 70% threshold volume.

To assess the gating+ accuracy, we generated 2,000 realizations of the moving lesion for each noise condition. There was a probability (*P* > 0.5) that the gating+ value was more accurate than the ungated value in every voxel in the simulations. We also found a probability (*P* > 0.5) of the gating+ value being more accurate than the gated value in 98% of the voxels. This last 2% occurred in voxels that had very high statistics, possibly because highest frequencies were not preserved in the gating+ method.

**Figure 6:**
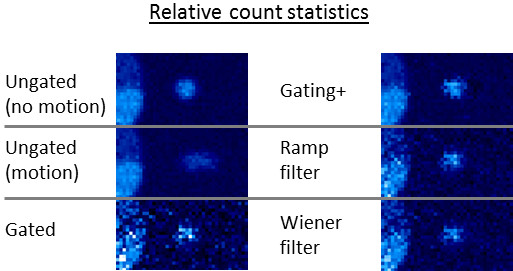
**Comparison of temporal filtering techniques using lesion simulation.** All filters are applied solely in temporal space.

To put the gating+ algorithm in perspective relative to other temporal filtering techniques, our simulation scenario was also processed using ramp and Wiener filters applied in the time domain, with results shown in Figure 6. The Wiener filtering approach we used is described by King and Miller [28].

In the preclinical PET scans, the maximum COM displacement for the population was 0.43 ± 0.41 mm (0.04 to 1.47 mm) (mean ± SD (range)) for the gating+ images. Kidney VOIs were definable for 52 scans, and liver profiles, 34 scans. A summary of the quantitative measurements is presented in Table 3. Shoulder/hip background VOIs were defined in all 84 scans. The average relative percent SDs in background regions for the gated, gating+, and ungated images were 1.51, 1.05, and 1.00, respectively, indicating superior noise presentation in ungated and gating+ images. All gating and gating+ measurements shown in Table 3 exhibited statistically significant effects (paired *t* test *P* < 0.01), indicating that gating+ had a measureable effect for all measurements. The filtering of high frequencies during the gating+ processing did not change the total activity value (summed over all gates) for any voxel - activity was conserved. The potential for introducing negative activity values does exist, but only in rare instances where the low-frequency fluctuations have a greater magnitude than the mean (DC) voxel value, and in these cases, it is only at minimal amounts. In the population of small-animal PET scans, 0.09% of voxels had negative values, and they were almost exclusively located in areas with no true activity, with a mean absolute negative activity of approximately 10^−10^ of the total scan activity.

**Table 3:** Preclinical PET motion effect measurements

			**Units**	**Measurements**			
				**Gated**	**Gating+**	**Gating+ with phase shift**	**Ungated**
Number of frames (phase increment = 360°/number)				16	16	90	1
Kidney VOI, *n* = 102	Average kidney uptake	Average VOI value, average of all gates	Relative values	1.01	1.01	1.01	1.00
	% SD	Average uptake across gates	Percentage	2.69	1.47	1.47	
	Maximum displacement	Average of all scans (SD of all scans)	mm	1.50 (0.56)	1.10 (0.75)	1.10 (0.75)	
Liver profile, *n* = 34	Liver boundary displacement		mm	3.25	3.04	3.05	
Shoulder VOI, *n* = 84	% SD in VOI	Average of all gates	Relative values	1.53	1.05	1.05	1.00
Global COM, *n* = 84	Maximum COM displacement	Average of all scans (SD of all scans)	mm	0.71 (0.46)	0.43 (0.41)	0.43 (0.41)	

Summary of VOI, line profile, and COM measurements in ungated, gated, and gating+ images across the population of preclinical PET scans. All numbers are average values of population or all appropriate population scans.

**Figure 7:**
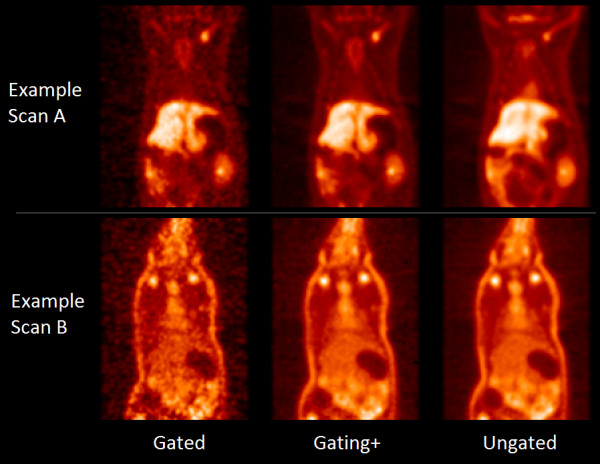
**PET images (coronal slice) illustrating differences in signal quality between gated, gating+, and ungated images.** In example A, the activity in the ribs is not well discernible from its surroundings in the gated image; however, in the corresponding gating+ image, the bones have discernible contrast. The gating+ image also shows better definition of the liver and kidneys. In example B, we again see discernible boundaries of the liver and ribs in the gating+ image and not in the gated image. Both images also highlight the global noise reduction provided by gating+. Example A was acquired with ^18^F-FDG, and example B was acquired with ^18^F-FBDPMA.

**Figure 8:**
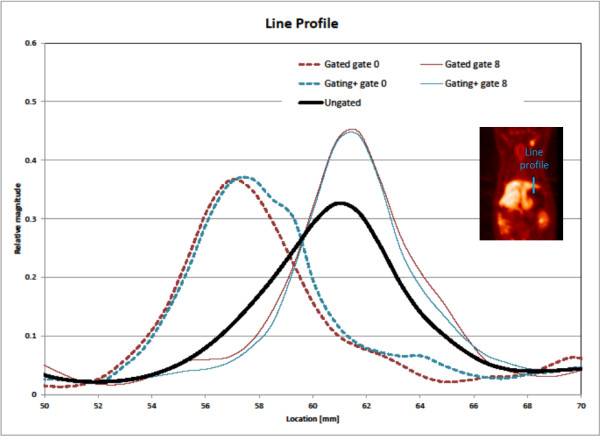
**Example motion profile.** Illustration of line profile placed on the rat shown in Figure 7, example A. Gated, gating+, and ungated profiles illustrate motion and spatial resolution characteristics.

An example small-animal PET image showing the noise differences between gated and gating+ images is shown in Figure 7. Resolution and motion effects for the rat shown in Figure 7 (example A) are illustrated with a sample line profile in Figure 8. It is seen that gated and gating+ images exhibit concentrations of activity at different locations in the different gates, an expected consequence of motion. The gated profiles have a slimmer width indicating improved image resolution relative to the ungated image. The change in the magnitude of activity concentration is an indication that activity is moving in three dimensions, traversing the coronal image plane. An image sequence, highlighting both noise and resolution/motion benefits resulting from processing, can be seen in Additional file 1.

### Phase-shifted CMI images

**Figure 9:**
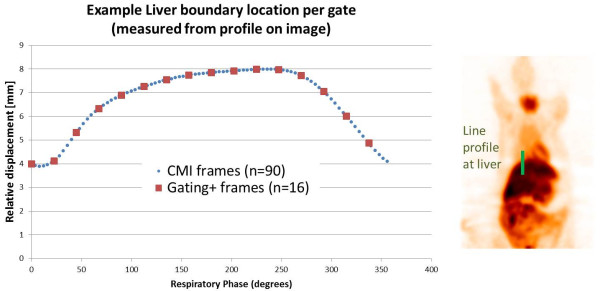
**Gate and CMI frame liver displacement measurements.** Image shows the liver dome location throughout the respiratory cycle as determined from a line profile placed on a small-animal PET image gated for respiratory motion. It is seen that the values derived from individual continuous motion image frames correlate well with the values from the gated image.

Phase-shifted images were generated to model continuous motion, with 90 frames/cycle. Figure 9 shows example liver boundary location measurements as determined from respective gates and CMIs. Table 3 shows that uptake measurements from these images were very similar to the gating+ images - kidney uptake measurements, liver displacement measurements, and background noise measurements averaged over the population were all within 1% of each other for the gating+ images and CMIs. Samples of the continuous motion image sequences can be seen in Additional files 2 and 3. A side-by-side comparison of corresponding gated and gating+ embodiments of selected scans with different levels of useful motion information is shown in Additional file 4, illustrating how the algorithm passes useful information while filtering noise.

To gain insight into the accuracy of the CMI measurements, simulations were constructed using randomly generated time-activity curves. Voxel activity vs. phase curves were generated randomly with signals in frequencies <Nyquist frequency. Gated (step function) values were derived from the true curves; CMI values were derived from gated values and evaluated. In 100% of the simulations (10^6^), the CMI curves correlated better with true motion curves than the respective gated curves.

The time required to process a 10-min acquisition from listmode, generating ungated, gated, and gating+ images, was approximately 1.5 h using a standard PC. Because the software we used in this study is proprietary, we were not able to optimize this processing. However, in our previous work [18], we showed that this processing time can be drastically reduced, to approximately the time of scan acquisition or better, if integrated with vendor software. The gating+ algorithm required approximately 20 s of processing once the requisite source images were available. Phase-shifted images required approximately 0.02 s/2D image slice to generate.

## Discussion

In this study, we have shown that data-driven gating methods previously demonstrated in human PET can be extended to small-animal PET and used with a large scan population, with diverse radiotracers and activity distributions. Furthermore, to generate less noisy gated images, we developed an algorithm based on voxel-specific temporal filtering to yield optimized gated images, denoted as gating+. The two techniques can be used separately or combined together into a motion correction workflow as we have done here. Both algorithms utilize motion information that is inherently contained in PET data and use it to create optimized images.

Data-driven and hardware-based gating are two very different approaches for acquiring essentially the same respiratory signature for a scan. Identical results cannot be expected from hardware and data-driven gating since hardware gating is based on signal recorded by a small device (in our case, an 18-mm-diameter pressure-sensitive pad placed under some part of the thorax of the rat), while the data-driven algorithm used in this study combines respiratory motion signals identified as periodic changes in activity concentration at any and all locations in the image volume. Nevertheless, in this work, visual and quantitative assessments confirmed that the two methods performed similarly with respect to trigger points and 4D image presentation for a subset of 24 scans, as has been observed by others [12,15]. In this assessment, however, we can note that there were two (/24) scans in which the median measured periods for these scans differed between hardware and software signal, and for which observers did find motion. Closer inspection revealed that for the first scan, both the hardware and software respiratory traces appeared very erratic, with significant portions of the scan interval exhibiting frequent large changes (>20%) in periodicity. Such behavior precludes useful respiratory gating, irrespective of the gating system. For the second scan, hardware gating yielded observable respiratory motion on the reconstructed images, while the software failed. Further scrutiny revealed that modifications to the frequency window parameter used in the software gating process could fix the error. This example highlights the importance of optimizing gating parameters and is discussed more below.

For the entire series of 84 scans, as expected, varying magnitudes of motion were observed in the gated and gating+ images, presumably reflecting different sizes of the rats and different levels of respiration. Our scan population also included some scans where the thorax and abdomen were almost entirely outside the axial FOV, and it was clearly appropriate that minimal, if any, respiratory motion should be detected in these scans. In such cases, there is no additional information to be gained from gating, yet there will be a cost in subdividing statistics. The combination of data-driven gating with gating+ signal optimization ensures that in such a case of inappropriate gating, the gating+ image will essentially default to its ungated state. This behavior is exemplified in Additional file 4.

For most PET scans, at least some respiratory information was captured - 75% of the scan population had motion confirmed by independent reviewers, and 71%, quantitatively using a global COM measurement. Liver profiles and kidney VOIs also identified characteristics of respiratory motion. On the other hand, we did not see significant change in SUV in the kidneys between gated and ungated scans, which may have been expected from analogous studies assessing lung lesions [21,34]. We suspect that kidney motion and uptake were not sufficient to show this effect.

In addition to extending software gating methods to preclinical PET, this work also addressed a fundamental consequence of gating: that gated images include less motion, but also fewer counts. This inherent trade-off presents a dilemma which inhibits the robust utilization of gating and provided the motivation to develop methods to improve the noise introduced in gated images.

The gating+ algorithm we presented is based on the filtering of signal in the temporal domain. In Figure 6, we can see a scenario where gated data were reconstructed using several filters: gating+, ramp, and Wiener. It is seen in this example that the gating+ algorithm is flexible enough to recover lesion resolution not present in the ungated image while also preserving background contrast not present in the gated image, and it handles this spectrum better than other filters, which provide more uniform filtering effects across the image. The other family of strategies used for addressing the poor statistics resulting from gating is based upon nonlinear motion mapping [22-24]. In an initial attempt to apply optical flow algorithms [22] to improve our gated images, the techniques appeared to work well for some scans yet performed prohibitively badly for others. One of the difficulties, common to these as well as other nonlinear image-morphing algorithms, lies in the need for calibrating definitions of signal and noise and in determining the freedom allowed in the mapping process. These parameters need to provide enough flexibility to achieve accurate results while avoiding the hazard of generating (potentially good-looking) images that are inaccurate. In diverse populations like that presented in this study, which include different noise levels, biodistributions, and FOVs, finding optimal algorithm parameters can prove difficult.

In contrast to motion mapping, the algorithm we are presenting is designed to avoid such problems, and all the available signals are optimized using a single one-dimensional temporal frequency filtering equation applied to the data for each voxel of the image volume. Scan-specific factors - complex activity distributions, poor scan conditions, variable noise levels - are all assessed and handled the same way. Since the algorithm is not dependent on scan conditions, it is characterizable, reproducible, and automated. Our software for creating gating+ images is built of about 15 lines of high-level (IDL) code, requires only seconds of processing per PET volume, and produces as output complete 4D image sets with associated frequency pass maps. Potential errors in gating+ images are limited by the fact that they are created with selective use of raw information. The fact that no higher frequencies are passed without supporting lower frequencies avoids the danger of unpredictable jumps in signal or Gibbs artifacts.

The implementation of the gating+ algorithm involves assessment of signal at every voxel in every frequency, selectively including the fluctuating signal due to respiratory motion only when and where it is not confounded by noise. To characterize the difference between useful signal and noise in a per-voxel per-frequency basis, we used an estimated threshold of 1.2× effective noise. The effective noise is derived from the randomly gated image. The constant 1.2 was derived from Monte Carlo simulations: 10^8 ^combinations of ranging magnitude and phase scenarios for motion and noise vectors were simulated. Both motion and noise vectors contain an element of noise which comes with random phase. We modeled this random process and found that when the ratio between motion and noise vector magnitudes is greater than approximately 1.2, then it becomes more probable (*P* > 0.5) that the gated signal is closer to the true signal than the ungated signal. In essence, the unknown phase of the noise is managed through knowledge of its magnitude, random phase, and statistical behavior, which allows us to make a binary determination as to its likely benefit on the accuracy of the gated signal. The concept may be understood as such: intuitively, where the true motion signal vectors are much greater in magnitude than the noise vectors, the gated signal is more reliable regardless of the noise and should be used. When the signal-to-noise ratio is poor, then useful fluctuations will be indiscernible through the noise, thus the gated signal provides no added value and should not be used. The implication of this strategy is that a gating+ voxel value will, on average, have improved accuracy relative to its ungated value.

When implementing gating, there is an important question of precision and accuracy of the motion capture. All forms of data-driven gating algorithms have several parameters which should be optimized to get the most favorable results: frequency pass windows, time bin duration, reconstruction parameters, etc. Then, the gating process too has parameters to be considered as well: trigger definition, data bin formation, number of histograms, etc. The significance of these issues were made clear to us when we found that changes in the frequency pass windows, used in the data-driven gating process, could affect the final results. However, changing the window to accommodate one scan degraded the quality of another, making it difficult to optimize the parameters.

Understanding this issue of what constitutes an optimal and non-optimal signal is of great importance as the field of gating moves forward in both the software-based and hardware-based arenas. Patient motion, uptake patterns, and scan statistics are very case specific. With hardware gating, results are variable with respect to the placement of monitoring devices and particular patient geometry/behavior, and parameter optimization in software gating can be understood as analogous. Our work demonstrated to us, however, that there may be a large advantage with software gating in that scans can be reprocessed retrospectively in an effort to achieve an optimal signal. There is potential for future methodological advancements in data-driven gating to incorporate iterative steps that will optimize all parameters during processing, making the algorithm more appropriate for use in diverse populations, possibly using the concept of a motion score [19]. Data-driven gating research could expand the classic concept of motion control achieved through gating towards algorithms that extract an optimal motion signal and present it with an optimal benefit.

While implementing the gating+ algorithm, we noticed some limitations/behaviors of the processing that we hope to address in future development of the algorithm. In voxels which have selective frequencies filtered, there is the potential to have a ‘shadow effect’ resulting from the fact that the true curve cannot be sufficiently modeled using the available lower-frequency sinusoidal waveforms passed in a band-pass filter. This effect can be seen in our simulations (Figure 2) where regions in the path of motion appear slightly darker than the background. We could not, however, find this effect in our preclinical images, likely because actual images have non-ideal statistical properties and deviate from perfect sinusoidal motion. However, because the gating+ voxel values are defined by the ‘optimal’ frequencies, they are still more likely to be accurate than the ungated values even if they are affected by this shadow. Future work can explore correcting this issue probably through a strategy of partial filtering in some frequencies, such as combining a Wiener filtration strategy with our approach for noise estimation, as opposed to the all-or-nothing band-pass approach we used here.

In addition to the images, the gating+ process creates a frequency pass ‘motion map’ that describes the distribution of motion information (Figure 5, Additional file 1). In future work, this map can potentially be used for motion characterization, lesion detection, gating optimization, or other gate utilization algorithms.

Also, we have begun to explore the ability for generating CMIs from 4D data. We are not creating any information to generate additional frames; rather, we are managing information that is available in a more flexible manner: we are considering gated data to define a step function in frequency space rather than in image space. While the gating+ processing is not a requirement for creating CMIs, the approach of optimizing signal in frequency space readily prepares the signal to be visualized at a user-defined phase while maintaining optimal statistics. The combined processes, illustrated in Figures 2 and 9, may offer intuitive presentations of motion, a new platform for understanding patterns in patient motion and organ-phase relationships, provide sub-gate activity derivatives which may be used for enhancing optical flow and/or other nonlinear mapping processes, and possibly present a new paradigm for understanding the trade-off between the signal/noise ratio vs. number of gates.

Motion control in nuclear medicine imaging currently remains a major obstacle impeding further resolution advancements. Despite a plethora of options to help address respiratory motion correction in PET, no clear optimal approach has emerged. Current commercial options for respiratory gating all use hardware which requires extra cost, time, effort, and training. Current gating research requires subjecting patients to additional scans [5]. In this work, we aimed to demonstrate that exclusively data-driven methodology for gating in PET is entering a new stage where software-based algorithms can create motion-corrected/noise-filtered scans in a fast and fully automated manner. Our methods use information that is present in the data and is not currently utilized. Signal optimization strategies like the one we are presenting provide a practical alternative for motion control in PET and may turn the long acquisition times required in nuclear medicine, traditionally considered a drawback, into a benefit.

## Conclusions

Data-driven gating and gating+ for image enhancement offer a strategy for creating motion-corrected images from ungated acquisitions that have noise characteristics similar to ungated images. The methodology was demonstrated on preclinical PET images with diverse activity distributions but should be equally applicable to clinical PET or other modalities. Future work will focus on improving methods and documenting clinical benefits of motion control. Data-driven gating and gating+ methods may be expanded to handle cardiac and other types of motion as well as other modalities, including SPECT/gamma camera imaging, CT, and ultrasound.

## Competing interests

Methods described here have been submitted for a US provisional patent.

## Authors’ contributions

ALK devised the project, developed the methods, helped with the image acquisition, processed the data, and drafted the manuscript. GA and EM were involved in the image acquisition, project design, and drafting of the manuscript. RS and ST were involved in drafting of the manuscript. NF acted as senior PI and aided with the project design, data acquisition, and drafting of the manuscript. All authors read and approved the final manuscript.

## Supplementary Material

Additional file 1: Figure S1 Example of a coronal slice from a small-animal PET rat scan. From left to right: summed (i.e., ungated) image, the same image, gated using software-based gating, same image with ‘gating+’ processing. On the right: motion map generated by the gating+ algorithm, where a higher signal indicates a higher cutoff frequency. Color scale is shown to the right of the motion map. Scan was reconstructed with 16 gates. Click here for file

Additional file 2: Figure S2 Example of an image slice. On the left, the image is shown in its gated embodiment. On the right, the same image is corrected using gating+ and shifted in phase to generate 90 frames distributed between 0 to 360°. Click here for file

Additional file 3: Figure S3 Examples of maximum-intensity projection images created using automated software gating, gating+ signal combination, and phase offset (30 frames/s) processes. Images displayed rotating 360° through cycle. Click here for file

Additional file 4: Figure S4 Central slices of eight small-animal PET scans are displayed in gated and gating+ form. Top four sets illustrate the gated and gating+ form of the scans which contain useful motion information. It is seen that the gating+ images maintain motion information while suppressing noise. The bottom four sets illustrate scans that do not benefit from gating. In these scans, we see that the reduction of image quality caused by gating is minimized in the gating+ images. Click here for file
