# Supplementary figures and images for: Gating, enhanced gating, and beyond: information utilization strategies for motion management, applied to preclinical PET
Source: EJNMMI Res. 2013 Apr 24;3:29. doi: 10.1186/2191-219X-3-29 (PMC3648448; doi:10.1186/2191-219X-3-29)

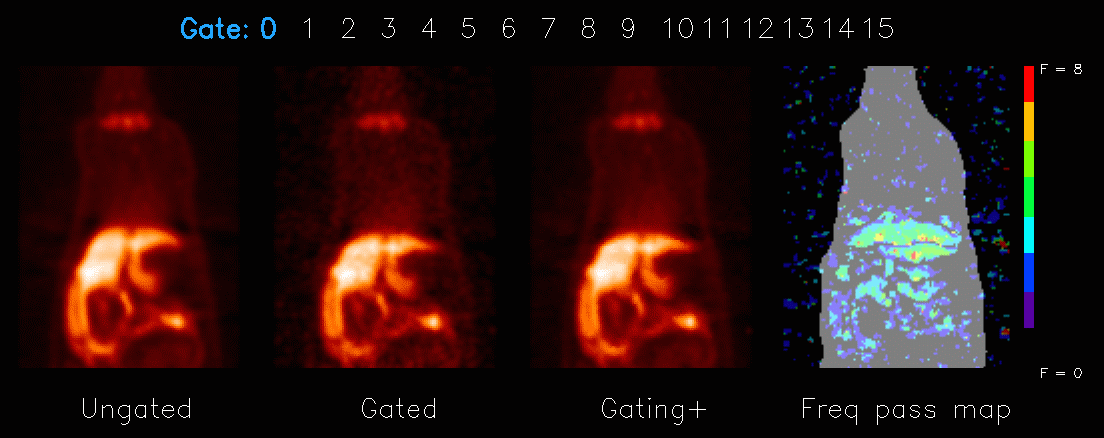

Supplement: Additional file 1: Figure S1 — Example of a coronal slice from a small-animal PET rat scan. From left to right: summed (i.e., ungated) image, the same image, gated using software-based gating, same image with ‘gating+’ processing. On the right: motion map generated by the gating+ algorithm, where a higher signal indicates a higher cutoff frequency. Color scale is shown to the right of the motion map. Scan was reconstructed with 16 gates. [file 2191-219X-3-29-S1.gif]
